# Strong genetic isolation of the black-lipped pearl oyster (*Pinctada margaritifera*) in the Marquesas archipelago (French Polynesia)

**DOI:** 10.1038/s41598-019-47729-w

**Published:** 2019-08-06

**Authors:** Céline Reisser, Cédrik Lo, David Schikorski, Manaarii Sham Koua, Serge Planes, Chin-Long Ky

**Affiliations:** 1 Ifremer, UMR EIO 241, Centre du Pacifique, BP 49, 98719 Taravao, Tahiti, French Polynesia; 2 Direction des Ressources Marines et Minières, BP 20, 98713 Papeete, Tahiti, French Polynesia; 3 Laboratoire Labofarm, 4 Rue Théodore Botrel 22603 Loudeac, Cedex, France; 4 PSL Research University: EPHE-UPVD-CNRS, USR 3278 CRIOBE, Labex Corail, Université de Perpignan, 52 Avenue Paul Alduy, 66860 Perpignan, Cedex, France

**Keywords:** Biodiversity, Population genetics

## Abstract

The French Polynesian islands are internationally known for their black pearls, produced by culture of the black lipped pearl oyster *Pinctada margaritifera*. The ongoing development of hatcheries for *P*. *margaritifera* in French Polynesia poses new challenges for the industry, particularly regarding the maintenance of genetic diversity in the hatchery stocks. This emphasizes the necessity to characterize the genetic diversity and differentiation within natural and exploited populations, to carefully select putative parental populations. The present study aimed at validating the phylogenetic status and investigating genetic attributes of populations from the only two non-exploited archipelagos of French Polynesia, the Marquesas archipelago, and the Australes archipelago, never analysed before. We found that individuals from both archipelagos belonged to *P*. *margaritifera* species. However, while the Australes population was genetically similar to non-exploited populations of the Tuamotu, the Marquesas populations were highly differentiated from the rest of the populations. This differentiation cannot not be only attributed to geographic distance and aquaculture status, but likely to hydrodynamic barriers allowing vicariant events to take place. Our results add up to other studies describing the Marquesas archipelago as a hotspot for biodiversity and differentiation, with some of the highest levels of endemism and vicariance found among marine species worldwide and provide precious information on available genetic resources for the implementation of *P*. *margaritifera* selective breeding and its genetic conservation in French Polynesia.

## Introduction

Located in the South Pacific Ocean, the French Polynesian islands consist of 118 atolls and high islands of volcanic origin distributed among five archipelagos: the Marquesas, Tuamotu, Society, Australes and Gambier archipelagos. These islands are separated by large bodies of open ocean (over 2000 meters of depth), and are considered as some of the remotest places on earth^1^. French Polynesia is internationally recognized for its black pearl industry and the aquaculture of the black-lip pearl oyster *Pinctada margaritifera*, and pearl farming sites are concentrated on 26 islands within three archipelagos: Tuamotu (80% of the production), Gambier (15%) and Society (5%).

The current exploited oyster stock originates entirely from natural spat collection through fixation of drifting larvae to a plastic substrate called a collector^2^. However, recent studies highlighting the interest of phenotypically selected hatchery produced oyster families for pearl quality and colour are now leading to the development of hatcheries of *P*. *margaritifera* in French Polynesia^3–5^. This poses new challenges for the industry, particularly regarding the maintenance of genetic diversity in the hatchery stocks. Indeed, many high fecundity marine organisms like oysters that underwent artificial breeding in the last few decades have shown a declining trend in genetic diversity within their broodstock^6,7^, as for hatchery produced *P*. *margaritifera* in Fiji^8^. This phenomenon is mostly explained by the high variance in reproductive success of oysters combined with high genetic load in hatchery-spawned pearl oysters, which can result in severe inbreeding depression and severely limit the long-term viability of selective breeding for a given species^9–11^. In addition, outbreeding depression can also act on the viability of a hatchery stock^12^. Outbreeding depression occur when the progeny from a cross between two genetically distant individuals exhibit lower fitness than its parents. It is thus extremely important to characterize the existing genetic diversity and levels of differentiation within natural and exploited populations, and carefully select putative parental populations in order to maintain similar levels of genetic diversity, effective population sizes, and fitness levels in hatchery families.

In French Polynesia, aquaculture of *P*. *margaritifera* is concentrated in 26 islands belonging to three archipelagos: Tuamotu (80% of the production), Gambier (15%) and Society (5%). Previous genetic investigation of farmed and wild populations of *P*. *margaritifera* from the three exploited archipelagos revealed that translocations have impacted the genetic variability of the species: while natural spat collection promotes high levels of genetic diversity in the collected stock and thus the farmed populations, the translocation of this collected stock across different islands led to a genetic homogenization (reduction in the levels of genetic differentiation) of the farmed stocks across entire archipelagos^7^. Indeed, after 10 years of translocations, the genetic differentiation among exploited archipelagos and among populations within exploited archipelagos decreased from 0.032 to 0.006 and from 0.025 to 0.007 respectively, regrouping the three archipelagos together into one genetic unit^13,14^. In addition, farmed individuals reproduce with adjacent natural populations creating genetic leakage and homogenization of the natural populations within exploited lagoons as well^7^.

**Figure 1:**
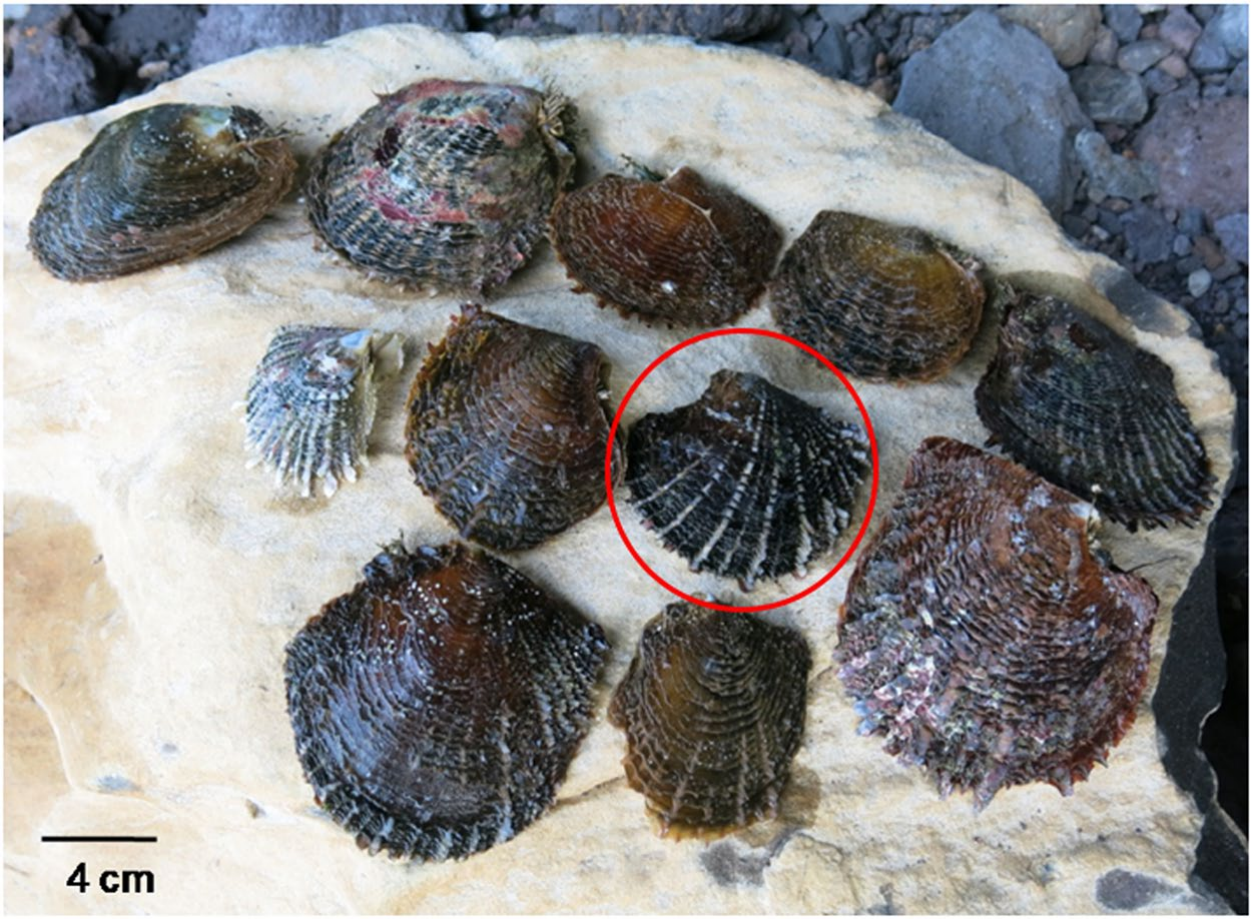
Phenotypic variability of wild *P*. *margaritifera* individuals collected from the non-exploited island of Ua Pou (Marquesas archipelago), showing a large diversity of shell coloration, never observed in the homogeneous black shell farmed population of the Tuamotu, Gambier and Society archipelagos (the typical black phenotype indicated by the red circle). The scale bar represents 40 mm.

To date, the Australes and the Marquesas archipelagos have never been exploited by the pearl industry, so their oyster populations remain unaffected by the homogenizing effect of translocations or genetic “leakage”. While no genetic investigation was carried on Australes populations, previous genetic investigation of the Marquesas populations (from Eiao and Hatutu islands) showed high levels of differentiation from the rest of the French Polynesian populations, attributed the recurrent isolation and vicariance found for the marine fauna of the Marquesas Islands combined with the absence of local aquaculture development, preserving the original stock from contamination by translocation^15^. While this genetic peculiarity could be useful for the maintenance of the diversity of hatchery populations, their habitat specificities and their phenotypic characteristics put into question the affiliation of the Marquesas populations to the species *margaritifera*, and their possible use as a source population for hatchery stock production. While *P*. *margaritifera* individuals are usually found in calm oligotrophic lagoon waters, they were found in open sea coastal shelves in the Marquesas archipelago^15^. A recent exploration of two other islands (Ua Pou and Nuku Hiva islands) also reported the presence of individuals in small intertidal tide pools subjected to temperatures that could reach 34 °C during the day (C.-L. Ky, unpublished data), a temperature that causes severe metabolic depression in individuals from the North Tuamotu^16^. In addition, the Marquesas’ *P*. *margaritifera* present a red/orange overtone of the shell and narrow spaced growth barbs, which diverges from the grey/black shell and wider spaced growth barbs phenotype of the rest of French Polynesia (Fig. 1). *P*. *margaritifera* is known to be polyphyletic, with one genetic clade from Mauritius (possibly representing a different species), and a second genetic clade grouping individuals from French Polynesia and Japan^17^. The polyphyletic status of *P*. *margaritifera* was actually previously suspected based on its wide geographic distribution and its intraspecific phenotypic polymorphism^18,19^, which further warrants our interrogation on the affiliation of the Marquesas samples. In addition, a recent investigation revealed that *P*. *mazatlanica* specimens, *P*. *margaritifera*’s sister species, has a basal position with regard to the Polynesian clade of *P*. *margaritifera*^20^. Early taxonomic classification of *P*. *mazatlanica* based on morphological characters had in fact classified it as a subspecies of *P*. *margaritifera* (*P*. *margaritifera mazatlanica*; since its shell morphology appeared to be an intermediate form between *P*. *margaritifera* and *P*. *maxima*^19,20^. The morphologic and life history divergence of the Marquesas samples from the other populations raises questions on their affiliation to the *P*. *margaritifera* species.

**Figure 2:**
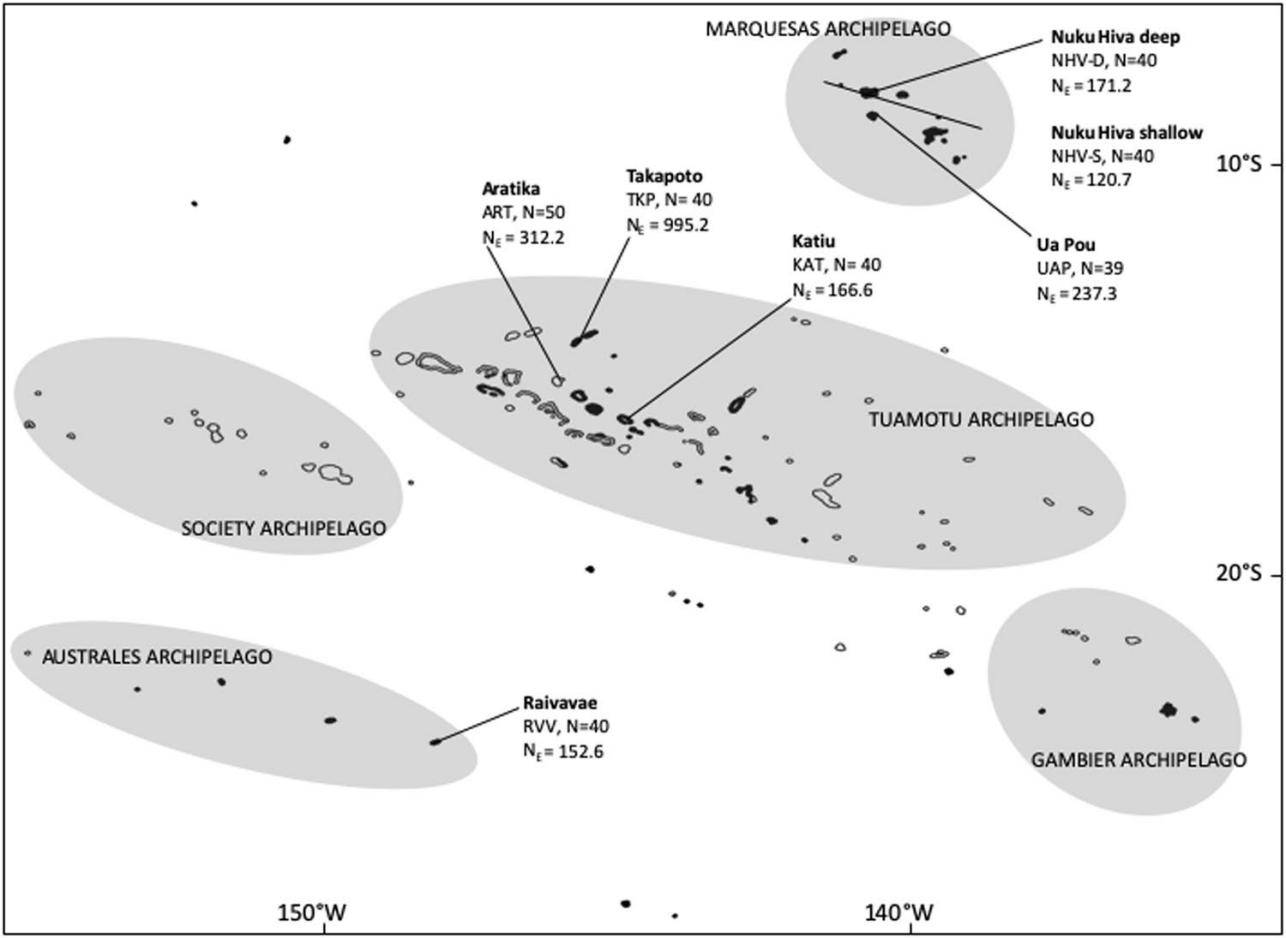
Sampling locations in French Polynesia where *P*. *margaritifera* specimens were collected. N: number of individuals sampled, N_E_: effective population size. GPS coordinates: NHV-D and NHV-S (08°50′38.6880″S/140°08′38.2380″W), UAP (09°22′51.9492″/140°04′26.5368″W), ART (15°32′34.7172″S/145°31′54.6852″W), TKP (14°37′41.2320″S/145°12′16.3332″W), KAT (16°25′30.6732″S/144°22′08.7888″W), RVV (23°51′44.8236″S/147°39′38.0664″W).

The present study aims at investigating the general patterns of genetic diversity and differentiation, as well as confirming/infirming the phylogenetic affiliation of *P*. *margaritifera* samples obtained from two non-farmed French Polynesian archipelagos, the Marquesas and the Australes archipelagos, through a representative sampling of the different *P*. *margaritifera* habitats found in the Marquesas archipelago and the addition of samples from the Australes archipelago, never investigated before (Fig. 2). We will perform (i) a phylogenetic investigation of the genus at the scale of French Polynesia with the newly added populations to validate the phylogenetic affiliation of the Marquesas and the Australes populations to the Polynesian clade of *P*. *margaritifera*, (ii) assess levels of genetic diversity in the Marquesas and the Australes archipelagos, and (iii) assess levels of differentiation of the Australes and the Marquesas archipelago from other farmed and non-farmed atolls of the Tuamotu. Ultimately, we aim at providing estimates of genetic diversity and differentiation of populations from two pristine archipelagos, to assist in the current development of a hatchery production in French Polynesia.

## Results

### Phylogenetic analysis

**Figure 3:**
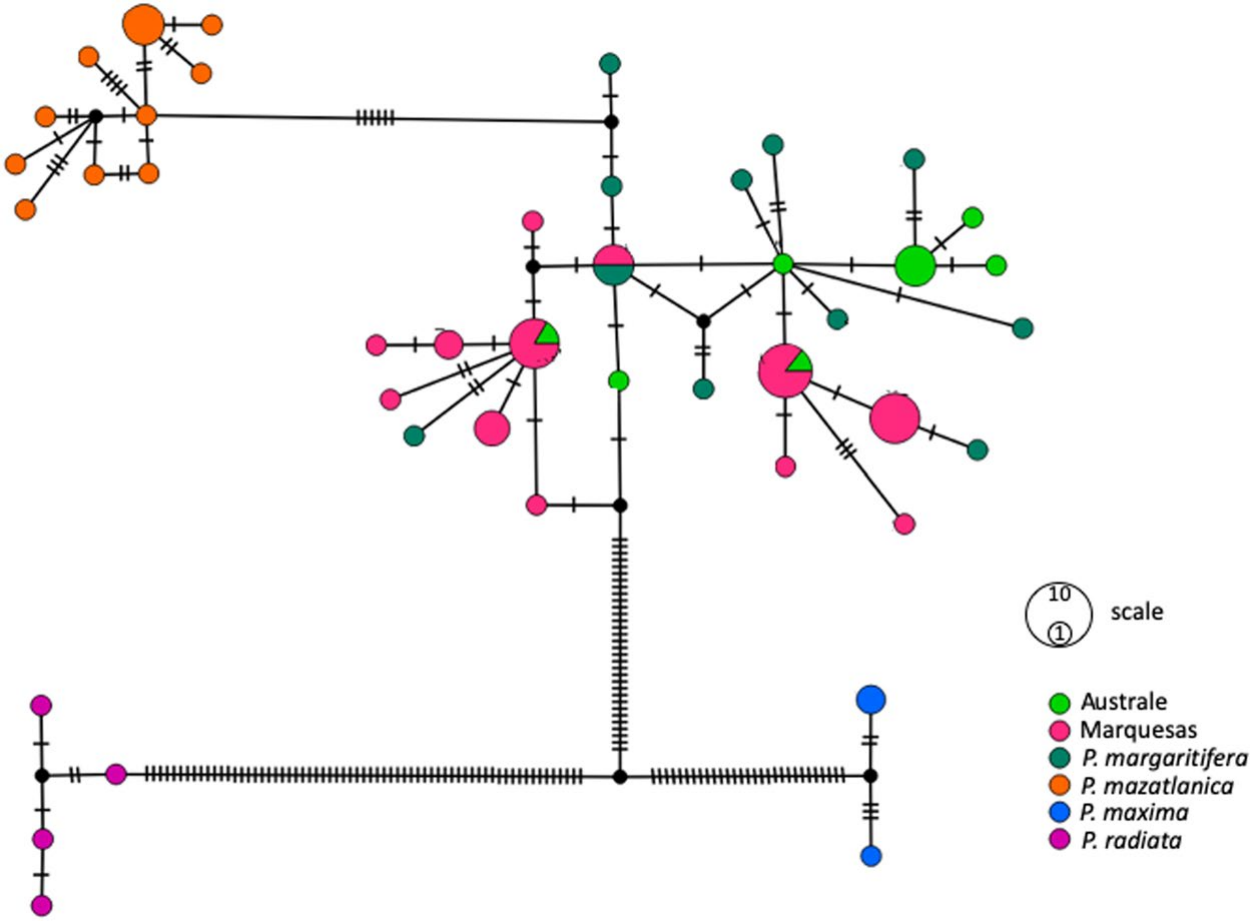
Haplotype network based on COI alignment of the *Pinctada* individuals using the TCS network algorithm in PopART.

We obtained a 422 bp long alignment of the COI sequences from all the 74 individuals used in this study. MCMC chains successfully converged (Effective Sample Size = 855.5). Bayesian phylogenetic analysis yielded the topology depicted in Supplementary Fig. S1. *Pinctada fucata*, *P*. *radiata* and *P*. *maxima* were all separated from the rest of the samples with a Bayesian Posterior Probability (BPP) of 100%. The Mauritius *P*. *margaritifera* sample have a basal position with respect to all the other *P*. *margaritifera* individuals with a BPP of 100% (Supplementary Fig. S1). Overall, the lack of statistical support and the random distribution of the Australe and Marquesas haplotypes with those from the rest of French Polynesia indicate an absence of geographical structuring for the COI gene. The haplotype network is congruent with that statement, with individuals from the Marquesas and the Australes populations are evenly distributed in the different *P*. *margaritifera* clusters, indicating an absence of geographical structuring for the COI gene (Fig. 3). In addition, there was no haplotype sharing between *P*. *margaritifera* and the other *Pinctada* species.

### Genetic diversity, population structure and isolation by distance

Genetic diversity estimates are presented in Table 1 (and Supplementary Table S1). We see that allelic richness is variable across loci, ranging from 6.329 for Pmarg68 to 20.557 for Pmarg45. All loci but Pmarg7 showed significant departure from HWE (Table 1A). Allelic richness (Ar) was the lowest in the Marquesas archipelago (ranging from 12.8 to 13.6), while the Australes population have the highest value (14.7). The estimated frequency of null alleles ranges from 0.140 for Katiu to 0.196 for Ua Pou, and is similar across all archipelagos (Table 1B). Heterozygosity levels (expected, observed and corrected) are high (above 0.4 for many populations) and are similar to those reported in previous studies of *P*. *margaritifera* populations from French Polynesia^7,15^. All the populations show *F*_IS_ values significantly different from 0, and deviation from HWE with excess of homozygotes (Table 1B).

**Table 1:** Diversity statistics of (A) the 9 loci genotyped and (B) the seven populations sampled.

Locus	Ar	N_GEN_	Freq_Null_	HE	HO	uexp_HET_	F_IS_	95% CI F_IS_	HWE
**(A)**									
Pmarg2	19.043	272	0.268 ± 0.061	0.919 ± 0.003	0.406 ± 0.117	0.931 ± 0.004	**0**.**558** ± **0**.**128**	0.410–0.686	significant
Pmarg7	9.343	288	0.106 ± 0.029	0.775 ± 0.078	0.585 ± 0.091	0.785 ± 0.079	**0**.**247** **+** ± **0**.**077**	0.078–0.396	not significant
Pmarg11	17.086	284	0.165 ± 0.040	0.891 ± 0.013	0.582 ± 0.080	0.902 ± 0.012	**0**.**347** ± **0**.**087**	0.195–0.479	significant
Pmarg37	17.000	288	0.146 ± 0.076	0.885 ± 0.023	0.613 ± 0.137	0.896 ± 0.023	**0**.**303** ± **0**.**172**	0.159–0.437	significant
Pmarg44	9.429	251	0.231 ± 0.067	0.754 ± 0.057	0.349 ± 0.109	0.765 + 0.057	**0**.**533** ± **0**.**148**	0.351–0.691	significant
Pmarg45	20.557	280	0.125 ± 0.045	0.916 ± 0.012	0.673 ± 0.082	0.928 + 0.013	**0**.**264** ± **0**.**096**	0.127–0.385	significant
Pmarg68	6.329	265	0.177 ± 0.081	0.646 ± 0.094	0.354 ± 0.136	0.655 ± 0.095	**0**.**457** ± **0**.**190**	0.257–0.639	significant
Pmarg77	14.214	241	0.282 ± 0.084	0.888 ± 0.044	0.361 ± 0.184	0.902 ± 0.043	**0**.**600** ± **0**.**195**	0.444–0.732	significant
Pmarg79	10.743	288	0.051 ± 0.032	0.782 ± 0.081	0.699 ± 0.057	0.791 ± 0.082	0.102 ± 0.076	−0.052–0.287	significant
**Pop**	**Ar**		**Freq**_**Null**_	**H**_**O**_	**H**_**E**_	**uexp**_**HET**_	**F**_**IS**_	**95% CI F**_**IS**_	**HWE**
**(B)**									
NHV-D	13.6		0.175 ± 0.113	0.485 ± 0.201	0.810 ± 0.107	0.821 ± 0.109	**0**.**399** ± **0**.**238**	0.334–0.440	significant
NHV-S	12.8		0.148 ± 0.098	0.524 ± 0.198	0.781 ± 0.146	0.792 ± 0.148	**0**.**330** ± **0**.**230**	0.261–0.375	significant
UAP	13		0.196 ± 0.109	0.453 ± 0.215	0.795 ± 0.114	0.807 ± 0.115	**0**.**431** + **0**.**265**	0.375–0.465	significant
ART	14		0.191 ± 0.096	0.513 ± 0.185	0.866 ± 0.063	0.875 ± 0.064	**0**.**407** ± **0**.**214**	0.345–0.454	significant
TKP	14.2		0.162 ± 0.066	0.552 ± 0.124	0.856 ± 0.081	0.867 ± 0.081	**0**.**355** ± **0**.**135**	0.293–0.393	significant
KAT	14		0.140 ± 0.073	0.580 ± 0.118	0.842 ± 0.100	0.853 ± 0.101	**0**.**305** ± **0**.**148**	0.241–0.344	significant
RVV	14.7		0.194 ± 0.097	0.488 ± 0.188	0.849 ± 0.093	0.860 ± 0.094	**0**.**427** ± **0**.**207**	0.365–0.467	**significant**

Ar: allelic richness, N_GEN_: number of individuals successfully genotyped, Freq_NULL_: estimated frequency of null alleles, H_O_: observed homozygosity, H_E_: expected heterozygosity, uexp_HET_: unbiased expected heterozygosity, HWE: p-value of Hardy-Weinberg Equilibrium test, F_IS_: inbreeding coefficient; 95% CI F_IS_: 95% confidence interval for F_IS_.

Boldface represent significance at alpha = 0.05.

F_ST_ and Jost’s D values are significantly different from 0 between the Marquesas archipelago and all the other populations, while they are not significantly different from 0 between the Australes archipelago and the Tuamotu archipelago (Table 2). F_ST_ values are not significant among the Marquesas populations and among the Tuamotu populations. Interestingly, Jost’s D values are significant between NHV-D and the two other Marquesas populations, NHV-S and UAP, and also between the two non-exploited populations of the Tuamotu, ART and KAT.

**Table 2:** Population genetic structure of the different *P*. *margaritifera* populations.

	NHV-D	NHV-S	UAP	ART	TKP	KAT	RVV
NHV-D		**0**.**0454**	**0**.**0472**	**0**.**1576**	**0**.**0957**	**0**.**1063**	**0**.**1512**
NHV-S	0.0085		0.0239	**0**.**2286**	**0**.**1286**	**0**.**1476**	**0**.**2113**
UAP	0.0087	0.0026		**0**.**2738**	**0**.**1681**	**0**.**182**	**0**.**1833**
ART	**0**.**0316**	**0**.**0427**	**0**.**0432**		0.0482	**0**.**0668**	0.0438
TKP	**0**.**0192**	**0**.**0338**	**0**.**0337**	0.0043		0.0417	0.0313
KAT	**0**.**0265**	**0**.**0328**	**0**.**0353**	0.0091	0.0071		0.0469
RVV	**0**.**0276**	**0**.**0426**	**0**.**0394**	0.0076	0.0051	0.0095	

Lower triangle values correspond to the *F*_ST_ values, while the upper triangle gives the Jost’s D values. Boldface indicate significance at 95% confidence intervals after 10000 bootstrap resampling.

**Figure 4:**
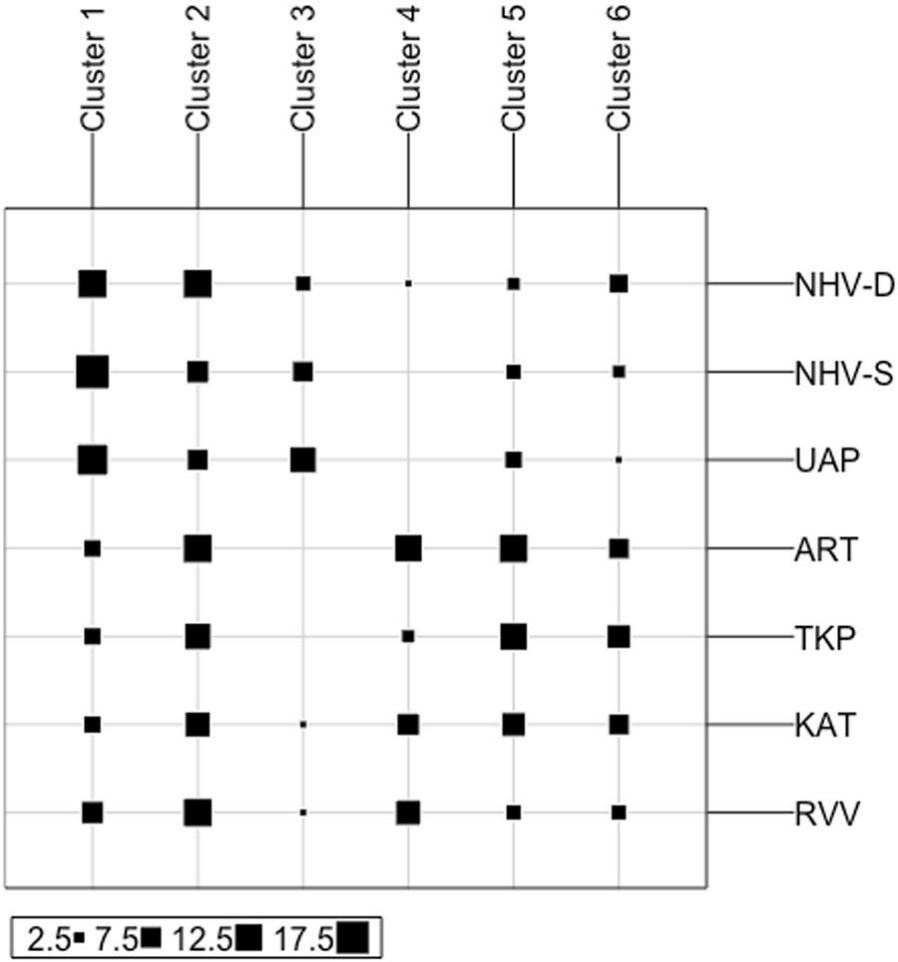
Results of the non-supervised hierarchical clustering that identified six clusters in our dataset: assignation of individuals from the 7 islands sampled to the six genetic clusters.

The non-supervised hierarchical clustering indicates the presence of six genetic clusters in the dataset (Fig. 4, Supplementary Fig. S2). While cluster 2 and cluster 6 contain a comparable number of individuals from all populations of all three archipelagos, cluster 3 and cluster 4 almost exclusively contain individuals from the Marquesas populations and the Tuamotu + Australes populations respectively (Fig. 4B). Cluster 1 and cluster 5 also show a preferential distribution biased toward individuals from the Marquesas for cluster 1, and toward individuals from the Tuamotu for cluster 5 (Fig. 4). The Australes individuals do not segregate preferentially within a particular cluster and can be found mostly alongside samples from the Tuamotu archipelago.

**Figure 5:**
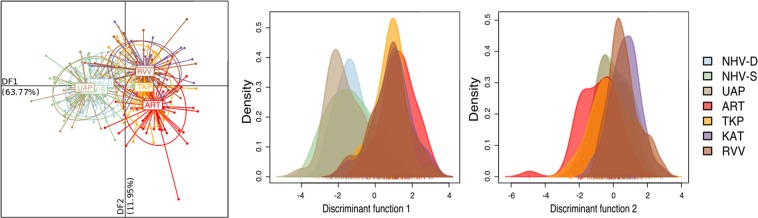
DAPC results: (left) scatterplot of the populations showing the first two discriminant axes; (centre) density plot of the distribution of each population on the first discriminant axis; (right) the density plot of the distribution of each population on the second discriminant axis.

**Figure 6:**
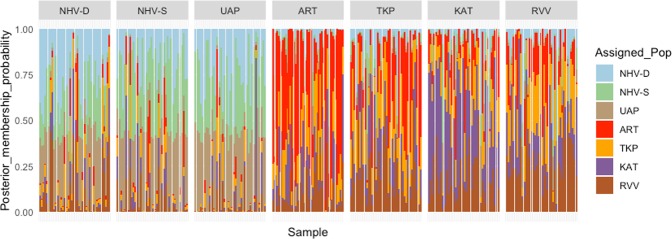
Composition plot (STRUCTURE-like plot) showing the probability of assignment of each *P*. *margaritifera* individual to the different populations.

The DAPC performed according to geographical location separates most of the Marquesas individuals from the rest of the populations along discriminant axis 1, which accounts for 63.77% of the total variance of the dataset (Fig. 5). Discriminant axis 2 imperfectly separates ART from the rest of the Tuamotu and Australes populations, and accounts for 11.95% of the total variance (Fig. 5). The composition plot also indicates that there is little exchange between the Marquesas archipelago and the other Polynesian archipelagos, with only a few individuals from the Tuamotu archipelago showing a possible affiliation (Fig. 6). As observed with the non-supervised clustering, individuals from the Australes archipelago however show similarity to individuals from the Tuamotu archipelago (Fig. 6).

**Figure 7:**
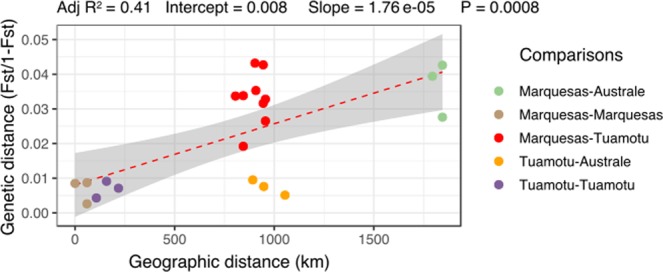
Scatterplot of the *P*. *margaritifera* genetic distance (populations pairwise F_ST_) as a function of the geographic distance (km). The red dotted line represents the fitted linear model describing the relationship between geographical distances and genetic differentiation. The grey shade represents the 95% confidence interval of the fitted values. R^2^ represents the amount of variability in the dataset explained by the linear fit.

Signal of isolation by distance is significant (Mantel test p-value = 0.015), with a positive correlation between geographical distance and genetic differentiation (Fig. 7; linear fit: R^2^ = 0.41, p-value = 0.008). However, for the distance of 1000 km, pairwise comparison involving the Marquesas populations show high levels of F_ST_, while pairwise comparisons involving the Australes population show F_ST_ levels similar to the intra-archipelagos one for the Marquesas and the Tuamotu populations (with less than 250 km separating the populations).

**Figure 8:**
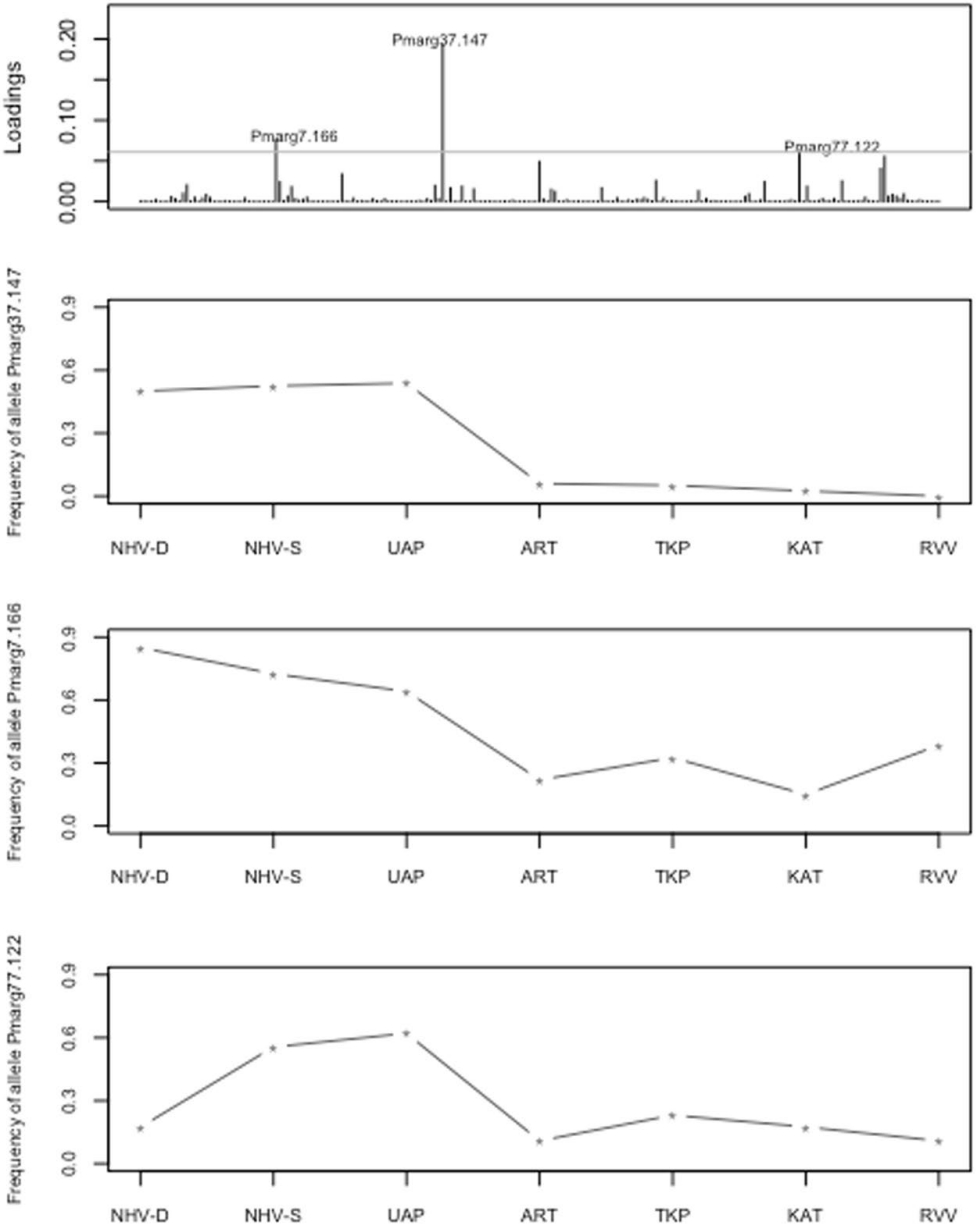
Loading plot showing the contribution of each microsatellite allele to the overall differentiation of the *P*. *margaritifera* samples (top) followed by line graphs of the variation in allelic frequencies of the top 3 alleles explaining the highest proportion of variance in the dataset.

The top 1 percentile alleles showing the greatest contribution to the differentiation found between all populations in the dataset contains three alleles (Fig. 8). Among these, the most discriminating is allele 147 of the Pmarg37 marker, which is present at a frequency of 50% and more in the Marquesas archipelago populations, but less than 10% in the other populations (Fig. 8). This allele segregates at both homozygous and the heterozygous states in all the Marquesas populations. Overall, all three alleles allow discrimination of the Marquesas samples from the rest of the dataset, displaying on average a higher frequency in the Marquesas samples (Fig. 8).

### Genetic relatedness, inbreeding and effective population size

**Figure 9:**
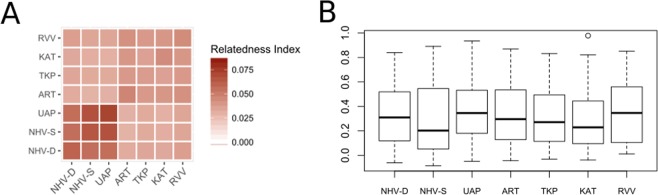
(**A**) Heatmap indicating the levels of relatedness of each *P*. *margaritifera* population; (**B**) boxplot showing the distribution of individual inbreeding coefficient for each *P*. *margaritifera* population.

Intrapopulation levels of relatedness are higher in the Marquesas than in other populations (Fig. 9A). The Australes population (RVV) and the two non-exploited populations of the Tuamotu, ART and KAT exhibits similar level of intrapopulation relatedness, but RVV differs from the exploited population TKP. There is a significant difference in interpopulation levels of relatedness (Kruskal Wallis p-value < 2.2E-16), which mostly occur between the Marquesas individuals and the rest of the samples, but also among populations within the Marquesas archipelago, with UAP showing differences in relatedness levels compared to NHV-D and NHV-S (Table 3). Also, relatedness levels of ART are different from that of TKP and KAT within the Tuamotu archipelago. Estimates of inbreeding are not significantly different among populations (p-value = 0.63; Fig. 9B). However, estimates of the effective population size N_E_ are highly variable according to the exploitation status of the populations: it ranges from 120.7 for NHV-S to 995.2 for TKP, the only exploited lagoon in our dataset (Fig. 2). All the non-exploited lagoons, including those of the Tuamotu, have a N_E_ < 313, three times less than TKP.

**Table 3:** Significance of the difference in the mean relatedness of *P*. *margaritifera* individuals among populations indicated by the Bonferroni corrected pairwise p-values of the Wilcoxon rank sum test (*p-value < 0.05; **p-value > 0.01; ***p-value < 0.001).

	NHV-D	NHV-S	UAP	ART	TKP	KAT	RVV
NHV-D	—						
NHV-S	0.2056	—					
UAP	2.00E-16***	2.00E-16***	—				
ART	0.0034**	1.00E-05***	2.00E-16***	—			
TKP	9.00E-10***	1.50E-13***	2.00E-16***	4.80E-05***	—		
KAT	1.50E-06***	9.00E-10***	2.00E-16***	0.0083**	0.2316	—	
RVV	2.40E-05***	4.20E-08***	2.00E-16***	0.0748	0.0444*	0.4074	—

## Discussion

Genetic conservation of aquatic resources, especially of exploited species is now an internationally recognized objective to sustainably manage and exploit marine populations, and to protect these populations/species^21^. In the present paper, we provide estimates of genetic diversity and differentiation for populations of pearl oysters from two pristine archipelagos to assist in the current development of a hatchery production for pearl production and insure sustainable use and genetic conservation of *P*. *margaritifera* in French Polynesia.

### Phylogenetic status and genetic diversity of the Marquesas and the Australe archipelagos

Phylogenetic analysis of COI mitochondrial gene reveals that populations of the Marquesas archipelago and the Australes archipelago group with all the other *P*. *margaritifera* samples from French Polynesia, validating their affiliation to the species, despite the extensive phenotypic and life history trait difference of the Marquesas individuals. We did not find a geographical clustering of the haplotypes, with both the Marquesas and Australe sharing similar haplotypes. Levels of genetic diversity are slightly lower in the Marquesas archipelago compared to the rest of the populations. Indeed, the archipelago contains populations with the lowest allelic richness and effective population sizes, while the level of relatedness among individuals was found to be the highest. This low genetic diversity cannot be attributed to ongoing inbreeding, since inbreeding coefficients were similar in all populations of our dataset, but could be the result of a founder effect (loss of genetic diversity that occurs when a new population is established by a very small number of individuals) and/or local selection, two processes that will be discussed below. Interestingly, genetic diversity indices of the Australe archipelago do not indicate a reduction in genetic diversity. The Australe population actually has a similar level of genetic diversity than that of the non-exploited Tuamotu populations, and shows the highest level of allelic richness, which reinforce the fact that the Marquesas populations are genetically peculiar at the scale of French Polynesia.

### Genetic differentiation of the Marquesas and the Australe populations

The Marquesas archipelago is genetically highly differentiated from both the Tuamotu and the Australe archipelagos. On the other hand, the Australe archipelago resembles the non-exploited populations of the Tuamotu, especially Katiu. While one could argue that geographic distance plays a role in levels of differentiation, the positive signal of isolation by distance we observed in our data set is clearly driven by the presence of the Marquesas populations (at equivalent geographical distance, the Australe population is not differentiated from the Tuamotu populations). This confirms that geographical distance per se is not the explanatory factor for the high levels of genetic differentiation of the Marquesas archipelago. There are thus two possible non-mutually exclusive scenarios that could explain the specificity of the Marquesas populations: selection, and a founding event. Lemer and Planes^15^ hypothesized that genetic differentiation of the Marquesas populations was correlated with a possible founder effect, followed by vicariance due to the presence of hydrodynamic barriers to connectivity. A founder effect and/or selection could also explain the unusual phenotype of the *P*. *margaritifera* individuals of the Marquesas archipelago. Indeed, the very common red/orange overtone of the outer shell of the Marquesas individuals (as opposed to the usual charcoal grey/black) does also occur at low frequency in some populations of the Tuamotu^22^, and could have been overly represented in the founding population.

Selection can also act on (and/or accelerate) genetic and phenotypic differentiation of the Marquesas individuals, considering the very different habitats of *P*. *margaritifera* in the Marquesas archipelago (open sea and intertidal warm tide pools). In these two extreme habitats, one might expect to find different selective pressures. However, despite the fact that the three populations of the Marquesas archipelago encompass both these extreme habitats, we did not find significant genetic differentiation among them. The lack of genetic structuring between the tide pool individuals and the open sea individuals seems to indicate no particular signs of selection for resistance to high temperature and hydrodynamic regimes. However, this result could also be due to the lack of power (through limited genome resolution) of our nine microsatellite markers to reveal a possible selection regime, likely acting on a small portion of the genome. Further analyses should be carried out with high density markers, such as SNPs, using both DNA sequencing and RNA Sequencing to target both expressed and non-expressed genomic regions (as performed and discussed in^8,23^).

### The Marquesas archipelago, an oasis in the middle of the pacific ocean

Because of their geographic isolation, oceanic islands have been the centre of interest of many biological monitoring for conservation biology and evolution^24^. Many species are endemic to these islands, and populations of non-endemic species are often found to be highly genetically differentiated from populations elsewhere. The Marquesas Islands are located at 1300 km from Tahiti (Society archipelago) and at 4800 km from Mexico, Central America being the closest continental landmass. This geographical isolation and particular geomorphological and hydrodynamic characteristics make the Marquesas archipelago a rich, dynamic and highly productive system that allow species to thrive. Indeed, numerous studies have reported high levels of genetic differentiation of the Marquesas archipelago marine and terrestrial populations compared to the rest of French Polynesia (sometimes to the rest of the Pacific) as well as the occurrence of a particularly high number of endemic species^25–29^. This high rate of endemicity and genetic diversity is thought to be partly attributed to the role of the Marquesas archipelago as a refuge during the Last Glacial Maximum (LGM)^15,30^, while subsequent genetic divergence is often generally explained by vicariance^15,27^).

Our results indicate that *P*. *margaritifera* individuals from the Marquesas archipelagos are indeed genetically divergent from the rest of the French Polynesian populations, with a higher differentiation than could be expected just by geographical isolation. Vicariance of *P*. *margaritifera* individuals might have taken place, alongside selection, considering their presence in new types of habitats. Our results thus add up to previously cited studies describing the Marquesas archipelago as a hotspot for biodiversity and differentiation, with some of the highest levels of endemism and vicariance found among marine species worldwide.

### Toward the development of hatchery populations of P. margaritifera

The present study allowed a better understanding of the distribution and levels of genetic diversity and differentiation in two non-exploited archipelagos in French Polynesia. It highlights the peculiarity of the Marquesas populations, which were deemed of great interest for hatchery production because of their possible temperature-specific adaptations. *Pinctada margaritifera* is already living close to the limit of its physiological optimum in most atolls of French Polynesia^16,31^, and there is thus a growing interest to understand how the oyster can adapt to rising temperatures. The breeding of the Marquesas populations could thus assist in maintaining temperature-resistant stocks of oysters in the face of global warming. However, care will have to be taken considering their high genetic divergence to the Tuamotu populations, which could result in outbreeding depression in the hatchery stock. Experimental crosses will have to be performed to test for this hypothesis.

Populations from the Australe archipelago were found to be genetically similar to the Tuamotu, and especially to the non-exploited atolls. This indicates that while the Tuamotu archipelago is mostly exploited and shows signs of genetic homogenization, non-exploited atolls might still carry a large amount of the original genetic diversity present in the Tuamotu before exploitation. Hence, these populations could represent the genetic baseline for the control of genetic diversity in hatchery populations.

## Materials and Methods

### Populations studied, tissue sampling and DNA extraction

For this investigation, seven populations of P. *margaritifera* were sampled (Fig. 2): three sites in the Marquesas (N = 119), three sites in the Tuamotu (N = 130) and one site in the Australes archipelago (N = 40). *Pinctada margaritifera* specimens between 8 and 20 centimetres in dorso-ventral shell measurements were collected (large size range to avoid cohort effects) (Fig. 8). In Nuku Hiva island, the pearl oyster population sampled by diving is referred to as NHV-D and the group sampled from the rocky shore in tide pools as NHV-S. In the Ua Pou (UAP) population, all samples were collected manually from the tide pools. A total of 289 individuals were sampled by taking a small piece of the mantle tissue, which was immediately transferred into 2.0 ml tubes containing 95° ethanol. The samples were stored at −20 °C until DNA extraction.

Genomic DNA was extracted from the mantle tissue (30 mg) using a KingFisher Flex automat (Thermo) and MagAttract®96 kit magnetic beads (Qiagen) following manufacturer’s recommendations. Briefly, a volume of 500 µL of magnetic beads was added to tissue samples previously lysed for 3 hours at 56 °C in deepwell plates containing 180 µL lysis buffer and 20 µL proteinase K. The magnetic bead-sample solution mixes were homogenized by shaking for 30 s. The DNA-beads complexes were then magnetically collected by the KingFisher magnets and washed twice with supplied washing solutions. DNA was finally released from the magnetic beads by incubation in 200 µL of elution buffer. Eluted DNA was stocked at −20 °C until use.

### Mitochondrial COI sequencing and quality check

In order to test for the phylogenetic status of the Marquesas and Australes populations, we sequenced the mitochondrial gene Cytochrome Oxidase I (COI), with the following primers: LCX 5′-TCG TAT AGA GCT CCG TCG ACC TG-3′ and HCY 5′-TGG AAC AAA ACT GGATCG CC-3′). We used PCR conditions similar to those used in^32^. We randomly selected 10 individuals per population in NHV-D, NHV-S, UAP and RVV and sequenced the 40 corresponding sequences on an ABI 3730XL (Applied Biosystems) automatic sequencer. The new sequences generated in this study were archived and are available in Genbank (NHV-D: MK913750 to MK913759, NHV-S: MK913760 to MK913769, UAP: MK913770 to MK913779, RVV: MK913740 to MK913749).

### Phylogenetic analysis

To perform the phylogenetic analysis of the *P*. *margaritifera* samples from the Marquesas and the Australes archipelago, we completed our sequence set with Genbank archived COI sequences of the 13 *P*. *margaritifera* individuals listed in^17^, from French Polynesia and Mauritius. To root *P*. *margaritifera*’s sequences, we also collected the Genbank archived COI sequences of 21 individuals from other *Pinctada* species present in the Pacific Ocean: *P*. *maxima*, *P*. *mazatlanica*, *P*. *fucata*, and *P*. *radiata*^17^.

We used Geneious V9.1.6^33^ to perform a global alignment of the 74 COI sequences using the MUSCLE algorithm with a maximum of 8 iterations. The global alignment obtained was curated manually to maximize positional homology. In order to obtain the optimal molecular model of evolution, we used the modeltest function in the R package phangorn^34^. The best fitting model was chosen according to the Akaike Information Criterion (AIC).

Phylogenetic reconstruction was performed using a Bayesian approach as implemented in MrBayes v3.2.5^35^. Four Metropolis-coupled Markov chain Monte Carlo (MCMC) analyses were run for one million generations and sampled every 100 generations, with a burn-in of 60000 generations. The GTR + G + I model of evolution selected by the Aikake Information Criterion in Modeltest. Trees were summarized through MrBayes and robustness was evaluated using Bayesian posterior probabilities (BPPs). The nexus file used in MrBayes is available in the Dryad repository (XXX). We used FigTree (http://tree.bio.ed.ac.uk/software/figtree/) to visualize the annotated tree. Finally, we built a haplotype network with the software PopART^36^, using the TCS haplotype builder algorithm.

### Microsatellite amplification, genotyping, and quality checking

For the diversity and hybridization status of the populations, nuclear DNA is preferred because of its biparental transmission and faster rate of evolution. *Pinctada margaritifera* samples were genotyped using a panel of nine microsatellite markers previously developed^37^. PCR reactions were performed using the Qiagen Multiplex PCR Kit (QIAGEN) in a 10 µL final volume containing 3 µL of genomic DNA diluted at 10 ng/µL, 5 µL Qiagen’s multiplex master mix, 1 µL Qiagen’s Q-solution, and 1 µL of an end-labelled primer mix. The nine selected microsatellite markers were amplified in 3 PCR multiplexes as detailed in Table 4. Amplifications were carried out in a GeneAmp PCR System 2700 thermal cycler (Applied Biosystems) following: initial denaturation at 95 °C for 15 min, followed by 35 cycles at 95 °C for 40 s, annealing temperature for 90 s, and elongation at 72 °C for 90 s, with a final extension at 60 °C for 30 min. Amplified PCR fragments were then diluted to a tenth with water and separated on an ABIPRISM 3130xl sequencer (Applied Biosystems) with GeneScan 500 Rox dye size standards.

**Table 4:** Microsatellite markers of *P*. *margaritifera*: primer sequences, amplification conditions, and allelic specificities (size range and count).

Multiplex	T*m*	Marker	Sequences (5′ to 3′)	Dyes	size range (bp)	Nb of alleles
Mix 1	56 °C	Pmarg37	F-GTCAGGATCTCCTTTATCTC	NED	145–195	17
			R-AGGAGATATGTCATTGCTG			
		Pmarg44	F-GGACAGGGAATATCAAAC	HEX	166–228	12
			R-CAAATATGTGCAGTGTGA			
		Pmarg77	F-GTTCAGCCATTCTTGAGAAG	FAM	118–188	19
			R-TGAGCAATATTTAGCTCGAAG			
Mix 2	55 °C	Pmarg2	FGATCCTACGATGATTGCTTTGTC	HEX	165–243	23
			R-TGCAACGTATCAGGTTATGTTTG			
		Pmarg11	F-TCTGTCCGTCCATCTAGC	NED	174–242	18
			R-ACAATGCATATCAAGTCAGC			
		Pmarg45	F-TCTGCCTGACAAGTTACGAAC	FAM	128–256	22
			R-ATACATTGAAGCTCGTCTCCTC			
Mix 3	50 °C	Pmarg7	F-CGTCAGTGGGAGTCAAATATTCG	HEX	160–188	11
			R-AGGAAGGGCATGTCATAAGGAAC			
		Pmarg68	F-GTTGCCTGTGAAACATAGTG	FAM	132–168	7
			R-CAGTTATGGCTGTGGACC			
		Pmarg79	F-AGTAAGTTGTAGCCAAATATGTGC	FAM	198–242	10
			R-GGAATATCAAACACAGGTCACTC			

Microsatellite alleles were visualized and scored using the GeneMapper v4.1 software system (Applied Biosystems, Life Technologies). Microsatellites were checked for repeat pattern accuracy and presence of null alleles in MICRO-CHECKER^38^.

### Genetic diversity, population structure and isolation by distance

To investigate the levels of genetic diversity within our samples, we calculated for each population and each locus the allelic richness, the observed, expected and corrected heterozygosity, F_IS_, and tested for HWE deviations with the diveRsity package^39^. F_IS_ significance was tested by calculating the 95% confidence intervals with 10000 bootstraps. We also used the diveRsity package to estimate genetic differentiation by calculating F_ST_ and Jost’s D for each pairwise population, and significance was tested with 10000 bootstraps. F_ST_ usually defines differentiation as a process mainly influenced by allele fixation through drift, while minimizing the role of mutation. Jost’s D on the other hand, gives equal weight to mutation and drift as potential processes leading to speciation. We used the software FreeNA^40^ to estimate the frequency of null alleles for each population at each locus.

To test for the presence of genetic structuring, we first used a non-supervised hierarchical clustering technique implemented in the find.clusters function in the adegenet package^41^, keeping 50 PC axes and using the ward method of clustering. The best number of clusters K was defined as the one minimizing the Bayesian Information Criterion (BIC). We then used a discriminant analysis of principal components (DAPC), a multivariate analysis that tries to maximize the variance among predefined groups of individuals (here geographical groups). To find the optimum number of PC axes to use without overfitting the data, we performed a cross validation using the xvalDapc function from adegenet (which defined 26 as the number of PC axes Achieving Lowest MSE). We then performed a DAPC with adegenet, followed by an individual-level assignment estimation for each population. To identify which alleles are driving the differentiation among populations, contribution of each allele to the global variance was visualized by extracting their loadings on the first discriminant axis.

Isolation by distance was tested using a matrix of geographic distance (in kilometres) obtained using a derivative of the great circle calculator, for latitude/longitude distance calculation (http://boulter.com/gps/distance/). Indeed, because we are working on small oceanic islands, the distance “at sea” between each pair is similar to that of a straight line between two GPS points. We used transformed F_ST_ estimates (F_ST_/(1-F_ST_)) as the measure of genetic distance between populations, and then correlated the genetic and geographic distance matrix using the function mantel.randtest in the ade4 R package^42^. Results of IBD were visualized in a scatterplot with a fitted regression line, obtained with the function lm of the stats R package.

### Genetic relatedness, inbreeding and effective population size

We used the coancestry function of the related package^43^ and the trioml method^44^ to calculate genetic relatedness among individuals within and between populations. The trioml method uses a maximum likelihood approach on a triad of individuals to estimate the relatedness index of two of them (pairwise comparison of two individual, with the addition of a control individual). This method supposedly makes a better estimate of true relatedness in natural populations (in which relatedness among two randomly chosen individuals would be negligible compared with within family relatedness estimation; see^40^ for further discussion of the method). Normality of the inbreeding coefficient and the relatedness indices was controlled with the Shapiro-Wilk test of normality through the shapiro.test function of the stats R base package. To examine whether any significant difference in the relatedness index among populations was correlated to a significant difference in levels of inbreeding among populations, we applied the Kruskal-Wallis test for multiple non-normal samples using the kruskal.test function of the stats R base package on both indices. For any significant difference among populations, the Wilcoxon rank sum test was used to identify the populations that were significantly different, and P-values were adjusted using the Bonferroni correction for multiple comparisons (function pairwise.wilcox.test in stats). Finally, we estimated N_E_ for each population using the program N_E_ESTIMATOR^45^, under random mating, using the bias-corrected version of the linkage disequilibrium method described in^46^, and considering only alleles with a Minor Allele Frequency (MAF) ≥ 0.05.

## Supplementary information

Supplementary Figures S1, S2 and Table S1

## Data Availability

The authors declare that all data are available. All sequences obtained in this study are available in Genbank (MK913740 - MK913779).
