# Supplementary material for: Strong genetic isolation of the black-lipped pearl oyster (Pinctada margaritifera) in the Marquesas archipelago (French Polynesia)
Source: Sci Rep. 2019 Aug 6;9:11420. doi: 10.1038/s41598-019-47729-w (PMC6684808; doi:10.1038/s41598-019-47729-w)
Supplement: Supplementary file 1 — Supplementary Figures S1, S2 and Table S1 [file 41598_2019_47729_MOESM1_ESM.docx]

**Supplementary information**

**Title of the manuscript:**

**Strong genetic isolation of the black-lipped pearl oyster (*Pinctada margaritifera*) in the Marquesas archipelago (French Polynesia).**

**Full author list:**

Céline REISSER, Cédrik LO, David SCHIKORSKI, Manaarii SHAM KOUA, Serge Planes, and Chin-Long KY*

**Supplementary Figure S1:**  Phylogenetic relationships of Pinctada pearl oysters from the Marquesas and Australes archipelagos based on a Bayesian inference (BI) analysis of mitochondrial COI sequence data using the GTR+I+Γ evolutionary model. Sequences from the species represented in bold were retrieved from GenBank. Numbers at the nodes correspond to Bayesian Posterior Probabilities (BPP). Only values above 70% are represented.


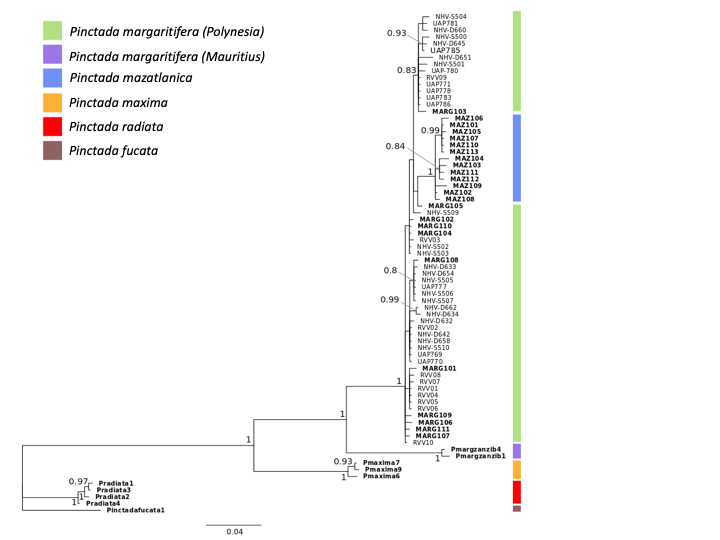


**Supplementary Figure S2**: BIC values of the non-supervised hierarchical clustering. The lowest BIC value obtained was for K=6 clusters.


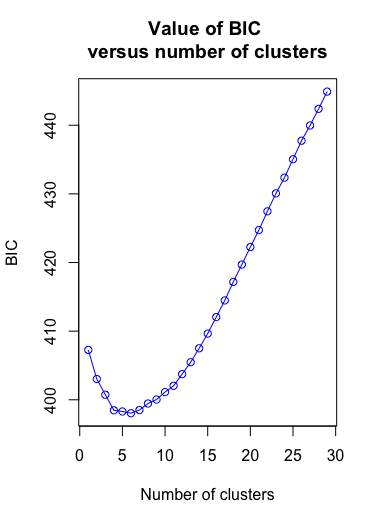


**Supplementary Table S1**. Diversity statistics of the seven populations sampled, at each of the 9 loci genotyped. Ar: allelic richness, NGEN: number of individuals successfully genotyped, FreqNULL: estimated frequency of null alleles, HO: observed homozygosity, HE: expected heterozygosity, uexpHET: unbiased expected heterozygosity, HWE: p-value of Hardy-Weinberg Equilibrium test, FIS: inbreeding coefficient; 95% CI FIS: 95% confidence interval for FIS. Boldface represent significance at alpha=0.05.

| **Pop** | **Locus** | **Ar** | **N_GEN_** | **Freq_Null_** | **H_O_** | **H_E_** | **uexp_HET_** | **HWE** | **F_IS_** | **95% CI F_IS_** |
| --- | --- | --- | --- | --- | --- | --- | --- | --- | --- | --- |
| **NHV-D** | Pmarg2 | 18.6 | 38.0 | 0.233 | 0.474 | 0.915 | 0.927 | **0.000** | **0.482** | 0.325 - 0.619 |
|  | Pmarg7 | 8.9 | 40.0 | 0.132 | 0.400 | 0.667 | 0.675 | **0.000** | **0.400** | 0.233 - 0.553 |
|  | Pmarg11 | 17.8 | 40.0 | 0.202 | 0.500 | 0.879 | 0.890 | **0.000** | **0.431** | 0.272 - 0.564 |
|  | Pmarg37 | 14.9 | 40.0 | 0.054 | 0.750 | 0.855 | 0.866 | 0.334 | 0.123 | -0.011 - 0.237 |
|  | Pmarg44 | 7.9 | 33.0 | 0.267 | 0.242 | 0.686 | 0.697 | **0.000** | **0.647** | 0.447 - 0.812 |
|  | Pmarg45 | 25.5 | 38.0 | 0.143 | 0.658 | 0.937 | 0.949 | **0.000** | **0.298** | 0.156 - 0.419 |
|  | Pmarg68 | 6.9 | 36.0 | 0.158 | 0.444 | 0.704 | 0.714 | **0.012** | **0.369** | 0.166 - 0.548 |
|  | Pmarg77 | 12.0 | 23.0 | 0.381 | 0.174 | 0.892 | 0.912 | **0.000** | **0.805** | 0.625 - 0.944 |
|  | Pmarg79 | 9.9 | 40.0 | 0.003 | 0.725 | 0.752 | 0.762 | 0.365 | 0.036 | -0.107 - 0.159 |
|  | overall | 13.6 | 36.4 | NA | 0.485 | 0.810 | 0.821 | **0.000** | **0.399** | 0.334 - 0.440 |
| **NHV-S** | Pmarg2 | 20.3 | 39.0 | 0.172 | 0.590 | 0.922 | 0.934 | **0.000** | **0.360** | 0.211 - 0.488 |
|  | Pmarg7 | 8.9 | 40.0 | 0.047 | 0.625 | 0.737 | 0.747 | 0.099 | 0.152 | -0.014 - 0.301 |
|  | Pmarg11 | 17.9 | 39.0 | 0.148 | 0.615 | 0.890 | 0.902 | **0.000** | **0.309** | 0.158 - 0.444 |
|  | Pmarg37 | 16.8 | 40.0 | 0.076 | 0.750 | 0.861 | 0.872 | **0.001** | 0.129 | -0.021 - 0.255 |
|  | Pmarg44 | 7.0 | 31.0 | 0.153 | 0.484 | 0.726 | 0.738 | **0.004** | **0.333** | 0.107 - 0.527 |
|  | Pmarg45 | 20.6 | 38.0 | 0.131 | 0.658 | 0.916 | 0.928 | **0.000** | **0.282** | 0.139 - 0.410 |
|  | Pmarg68 | 5.8 | 38.0 | 0.205 | 0.211 | 0.498 | 0.505 | **0.000** | **0.577** | 0.348 - 0.763 |
|  | Pmarg77 | 11.5 | 27.0 | 0.361 | 0.185 | 0.851 | 0.867 | **0.000** | **0.782** | 0.620 - 0.911 |
|  | Pmarg79 | 6.0 | 40.0 | 0.042 | 0.600 | 0.627 | 0.635 | 0.097 | 0.043 | -0.160 - 0.224 |
|  | overall | 12.8 | 36.9 | NA | 0.524 | 0.781 | 0.792 | **0.000** | **0.330** | 0.261 - 0.375 |
| **UAP** | Pmarg2 | 18.7 | 37.0 | 0.281 | 0.378 | 0.918 | 0.930 | **0.000** | **0.588** | 0.437 - 0.719 |
|  | Pmarg7 | 9.0 | 39.0 | 0.112 | 0.538 | 0.686 | 0.695 | **0.001** | 0.215 | -0.001 - 0.399 |
|  | Pmarg11 | 15.0 | 38.0 | 0.198 | 0.500 | 0.873 | 0.885 | **0.000** | **0.427** | 0.265 - 0.570 |
|  | Pmarg37 | 17.0 | 39.0 | 0.069 | 0.769 | 0.870 | 0.881 | **0.032** | 0.116 | -0.027 - 0.236 |
|  | Pmarg44 | 10.9 | 32.0 | 0.266 | 0.281 | 0.746 | 0.758 | **0.000** | **0.623** | 0.443 - 0.782 |
|  | Pmarg45 | 21.0 | 36.0 | 0.203 | 0.528 | 0.926 | 0.939 | **0.000** | **0.430** | 0.281 - 0.562 |
|  | Pmarg68 | 5.0 | 31.0 | 0.236 | 0.226 | 0.588 | 0.598 | **0.000** | **0.616** | 0.385 - 0.807 |
|  | Pmarg77 | 9.3 | 29.0 | 0.370 | 0.138 | 0.809 | 0.823 | **0.000** | **0.829** | 0.693 - 0.952 |
|  | Pmarg79 | 11.0 | 39.0 | 0.031 | 0.718 | 0.741 | 0.750 | 0.052 | 0.031 | -0.132 - 0.166 |
|  | overall | 13.0 | 35.6 | NA | 0.453 | 0.795 | 0.807 | **0.000** | **0.431** | 0.375 - 0.465 |
| **ART** | Pmarg2 | 19.5 | 46.0 | 0.342 | 0.261 | 0.917 | 0.927 | **0.000** | **0.715** | 0.591 - 0.825 |
|  | Pmarg7 | 10.6 | 50.0 | 0.130 | 0.620 | 0.838 | 0.847 | **0.006** | **0.260** | 0.110 - 0.391 |
|  | Pmarg11 | 16.5 | 48.0 | 0.195 | 0.542 | 0.913 | 0.922 | **0.000** | **0.406** | 0.268 - 0.529 |
|  | Pmarg37 | 16.6 | 50.0 | 0.233 | 0.460 | 0.908 | 0.917 | **0.000** | **0.494** | 0.359 - 0.616 |
|  | Pmarg44 | 10.4 | 47.0 | 0.201 | 0.447 | 0.811 | 0.819 | **0.000** | **0.449** | 0.297 - 0.585 |
|  | Pmarg45 | 19.1 | 50.0 | 0.074 | 0.760 | 0.900 | 0.909 | 0.204 | **0.155** | 0.041 - 0.257 |
|  | Pmarg68 | 6.7 | 48.0 | 0.270 | 0.271 | 0.742 | 0.750 | **0.000** | **0.635** | 0.488 - 0.770 |
|  | Pmarg77 | 16.7 | 46.0 | 0.235 | 0.478 | 0.930 | 0.940 | **0.000** | **0.486** | 0.346 - 0.612 |
|  | Pmarg79 | 9.5 | 50.0 | 0.036 | 0.780 | 0.834 | 0.842 | 0.727 | 0.064 | -0.054 - 0.173 |
|  | overall | 14.0 | 48.3 | NA | 0.513 | 0.866 | 0.875 | **0.000** | **0.407** | 0.345 - 0.454 |
| **TKP** | Pmarg2 | 20.6 | 38.0 | 0.288 | 0.368 | 0.923 | 0.935 | **0.000** | **0.601** | 0.453 - 0.730 |
|  | Pmarg7 | 9.0 | 40.0 | 0.122 | 0.625 | 0.845 | 0.855 | **0.000** | **0.260** | 0.100 - 0.397 |
|  | Pmarg11 | 15.8 | 40.0 | 0.109 | 0.700 | 0.898 | 0.909 | 0.214 | **0.220** | 0.076 - 0.347 |
|  | Pmarg37 | 19.0 | 39.0 | 0.183 | 0.564 | 0.912 | 0.924 | **0.000** | **0.382** | 0.230 - 0.514 |
|  | Pmarg44 | 7.5 | 35.0 | 0.192 | 0.371 | 0.702 | 0.713 | **0.000** | **0.471** | 0.276 - 0.639 |
|  | Pmarg45 | 18.7 | 40.0 | 0.136 | 0.650 | 0.912 | 0.924 | **0.042** | **0.287** | 0.145 - 0.415 |
|  | Pmarg68 | 6.6 | 38.0 | 0.142 | 0.500 | 0.761 | 0.772 | **0.009** | **0.343** | 0.162 - 0.500 |
|  | Pmarg77 | 18.1 | 39.0 | 0.216 | 0.513 | 0.930 | 0.942 | **0.000** | **0.448** | 0.297 - 0.582 |
|  | Pmarg79 | 12.9 | 40.0 | 0.068 | 0.675 | 0.823 | 0.833 | 0.076 | **0.180** | 0.040 - 0.303 |
|  | overall | 14.2 | 38.8 | NA | 0.552 | 0.856 | 0.867 | **0.000** | **0.355** | 0.293 - 0.393 |
| **KAT** | Pmarg2 | 18.6 | 39.0 | 0.226 | 0.487 | 0.915 | 0.927 | **0.000** | **0.468** | 0.311 - 0.604 |
|  | Pmarg7 | 9.0 | 40.0 | 0.102 | 0.675 | 0.854 | 0.865 | 0.080 | **0.210** | 0.055 - 0.345 |
|  | Pmarg11 | 17.8 | 40.0 | 0.186 | 0.550 | 0.895 | 0.907 | **0.000** | **0.386** | 0.229 - 0.522 |
|  | Pmarg37 | 17.8 | 40.0 | 0.204 | 0.500 | 0.893 | 0.905 | **0.000** | **0.440** | 0.293 - 0.572 |
|  | Pmarg44 | 9.6 | 38.0 | 0.191 | 0.421 | 0.762 | 0.773 | **0.000** | **0.448** | 0.271 - 0.601 |
|  | Pmarg45 | 18.5 | 40.0 | 0.071 | 0.775 | 0.910 | 0.921 | 0.406 | **0.148** | 0.020 - 0.256 |
|  | Pmarg68 | 7.5 | 39.0 | 0.022 | 0.538 | 0.608 | 0.616 | 0.777 | 0.114 | -0.074 - 0.286 |
|  | Pmarg77 | 14.7 | 40.0 | 0.186 | 0.550 | 0.896 | 0.907 | **0.000** | **0.386** | 0.230 - 0.519 |
|  | Pmarg79 | 12.9 | 40.0 | 0.075 | 0.725 | 0.848 | 0.859 | **0.031** | 0.145 | -0.009 - 0.279 |
|  | overall | 14.0 | 39.6 | NA | 0.580 | 0.842 | 0.853 | **0.000** | **0.305** | 0.241 - 0.344 |
| **RVV** | Pmarg2 | 17.0 | 35.0 | 0.331 | 0.286 | 0.921 | 0.934 | **0.000** | **0.690** | 0.540 - 0.815 |
|  | Pmarg7 | 10.0 | 39.0 | 0.099 | 0.615 | 0.798 | 0.809 | 0.094 | **0.229** | 0.066 - 0.372 |
|  | Pmarg11 | 18.8 | 39.0 | 0.114 | 0.667 | 0.889 | 0.900 | **0.007** | **0.250** | 0.103 - 0.377 |
|  | Pmarg37 | 16.9 | 40.0 | 0.202 | 0.500 | 0.893 | 0.904 | **0.000** | **0.440** | 0.291 - 0.572 |
|  | Pmarg44 | 12.7 | 35.0 | 0.350 | 0.200 | 0.845 | 0.857 | **0.000** | **0.763** | 0.616 - 0.890 |
|  | Pmarg45 | 20.5 | 38.0 | 0.117 | 0.684 | 0.912 | 0.924 | **0.005** | **0.250** | 0.108 - 0.376 |
|  | Pmarg68 | 5.8 | 35.0 | 0.208 | 0.286 | 0.624 | 0.633 | **0.000** | **0.542** | 0.326 - 0.726 |
|  | Pmarg77 | 17.2 | 37.0 | 0.228 | 0.486 | 0.910 | 0.922 | **0.000** | **0.465** | 0.296 - 0.607 |
|  | Pmarg79 | 13.0 | 39.0 | 0.100 | 0.667 | 0.848 | 0.859 | **0.039** | **0.214** | 0.058 - 0.355 |
|  | overall | 14.7 | 37.4 | NA | 0.488 | 0.849 | 0.860 | **0.000** | **0.427** | 0.365 - 0.467 |
